# Susceptibility breakpoint for Danofloxacin against swine *Escherichia coli*

**DOI:** 10.1186/s12917-019-1783-2

**Published:** 2019-02-04

**Authors:** Yuqi Yang, Yixin Zhang, Jiarui Li, Ping Cheng, Tianshi Xiao, Ishfaq Muhammad, Hongxiao Yu, Ruimeng Liu, Xiuying Zhang

**Affiliations:** 1 Heilongjiang Key Laboratory for Animal Disease Control and Pharmaceutical Development. Faculty of Basic Veterinary Science, College of Veterinary Medicine, Northeast Agricultural University, 600 Changjiang Road, Xiangfang District, Harbin, People’s Republic of China; 2 Department of Basic Veterinary Science, College of Veterinary Medicine, Northeast Agricultural University, Harbin, Heilongjiang 150030 People’s Republic of China

**Keywords:** Danofloxacin, *Escherichia coli*, ECV, CO_PD_, Monte Carlo simulation

## Abstract

**Background:**

Improper use of antimicrobials results in poor treatment and severe bacterial resistance. Breakpoints are routinely used in the clinical laboratory setting to guide clinical decision making. Therefore, the objective of this study was to establish antimicrobial susceptibility breakpoints for danofloxacin against *Escherichia coli* (*E.coli*), which is an important pathogen of digestive tract infections.

**Results:**

The minimum inhibitory concentrations (MICs) of 1233 *E. coli* isolates were determined by the microdilution broth method in accordance with the guidelines in Clinical and Laboratory Standards Institute (CLSI) document M07-A9. The wild type (WT) distribution or epidemiologic cutoff value (ECV) was set at 8 μg/mL with statistical analysis. Plasma drug concentration data were used to establish pharmacokinetic (PK) model in swine. The in vitro time kill test in our study demonstrated that danofloxacin have concentration dependent activity against *E.coli*. The PK data indicated that danofloxacin concentration in plasma was rapidly increased to peak levels at 0.97 h and remained detectable until 48 h after drug administration. The pharmacodynamic cutoff (CO_PD_) was determined as 0.03 μg/mL using Monte Carlo simulation. To the best of our knowledge, this is the first study to establish the ECV and CO_PD_ of danofloxacin against *E.coli* with statistical method.

**Conclusions:**

Compared to the CO_PD_ of danofloxacin against *E.coli* (0.03 μg/mL), the ECV for *E.coli* seemed reasonable to be used as the final breakpoint of danofloxacin against *E.coli* in pigs. Therefore, the ECV (MIC ≤8 μg/mL) was finally selected as the optimum danofloxacin susceptibility breakpoint for swine *E.coli*. In summary, this study provides a criterion for susceptibility testing and improves prudent use of danofloxacin for protecting public health.

## Background

*Escherichia coli* (*E.coli*) are a common member of microflora of the gastrointestinal tract of animals and humans. Pathogenic *E. coli* associated with gastrointestinal disorders have been divided into eight pathotypes based on their virulence profiles: enteropathogenic *E. coli* (EPEC); enterohaemorrhagic *E. coli* (EHEC); enterotoxigenic *E. coli* (ETEC); enteroinvasive *E. coli* (EIEC); enteroaggregative *E. coli* (EAEC); diffusely adherent *E. coli* (DAEC); adherent invasive *E. coli* (AIEC); and shiga toxin-producing enteroaggregative *E. coli* (STEC) [1]. Among them, ETEC infects both humans and several species of farm animals such as pigs. In humans, ETEC is the main cause of bacterial diarrhea in adults and children in developing countries and also a leading cause of traveler’s diarrhea [2, 3]. In pigs, enteric diseases due to strains of ETEC are the most commonly occurring form of colibacillosis including neonatal diarrhoea and postweaning diarrhoea (PWD), which result in significant economic losses due to mortality, morbidity, reduced growth rate and cost of medication [4].

Quinolones, which trap DNA gyrase or topoisomerase IV to form reversible drug enzyme DNA cleavage complexes, to cause bacteriostasis, have a high bioavailability, good tissue penetration, long half-lives, high efficacy, and low incidence of adverse effects. Because of these characteristics, they are widely used against several respiratory and gastrointestinal infections in both humans and animals [5, 6]. The antimicrobial treatment of traveler’s diarrhea has changed over the years because of the increasing resistance of ETEC to common antibiotics. So far, fluoroquinolones have been shown to be an effective therapy for ETEC traveler’s diarrhea [7, 8]. Danofloxacin (DANO), a third generation fluoroquinolone antimicrobial drug with rapid bactericidal activity, is often employed to treat colibacillosis in swine via oral or intramuscular administration [9, 10]. However, their extensive use has also serious non-desirable impacts and represents a public health danger. For example, it may stimulate the emergence of zoonotic quinolone-resistant *E.coli* in the food-producing animals, which can ultimately be transmitted to the human by direct contact or through the food chain [11, 12]. Plasmid-mediated fluoroquinolone resistance genes (qnrS and aac (6′)-Ib-cr) are detected in both patients and pigs in Shandong, China, and these resistance genes can be transmitted horizontally [13].

Improper use of antibiotics results in severe bacterial resistance. Breakpoints are routinely used in the clinical laboratory setting to guide clinical decision making. A combination of MIC values, pharmacokinetic/pharmacodynamic relationship and clinical outcome data are needed to set breakpoints [14]. However, this kind of data needed for breakpoint determination is so difficult and expensive to generate. Epidemiological cutoff values (ECVs) are the useful tools for laboratories conducting susceptibility testing and for clinicians treating infections. Those tools also offer alternative ways for monitoring the emergence of drug resistance in any given bacterial species [14]. ECVs establishment using the CLSI method must include MIC distributions (≥ 100 MIC results per species and Antibacterial agent) from multiple (≥ 3) independent laboratories [15, 16]. Previous studies demonstrated that a statistical method was a professional and scientific method which has been adopted by the CLSI as a standard method for ECV determination [17, 18]. Pharmacodynamic cutoff (CO_PD_) is associated with clinical efficacy, as both WT values and PK/PD data are used to setting CO_PD_ without clinical cutoff values [19]. The CO_PD_ was defined as the MIC at which the probability of target attainment (PTA) was ≥90% [20]. Monte Carlo simulation has been employed to assess the probability of attaining the desired AUC:MIC ratio, and it provides a means by which probability outcomes, such as achieving the PK/PD target, can be attained without the rigor, time, and expense of clinical trials [21, 22].

The purposes of the present study were (i) to develop ECV of DANO against *E.coli* using a statistical method and (ii) to establish DANO CO_PD_ for *E.coli* based on Monte Carlo simulation.

## Results

### Isolates

From July 2014 to March 2017, a total of 861 *E.coli* isolates were identified from 864 rectal/cloacal swabs of pigs. Isolates were collected from Heilongjiang (*n* = 296), Jilin (*n* = 151), Liaoning (*n* = 238), Henan (*n* = 97), Shandong (*n* = 30), Hubei (*n* = 20), and Yunnan (*n* = 29) provinces of China.

### Antibacterial susceptibility testing

**Fig. 1:**
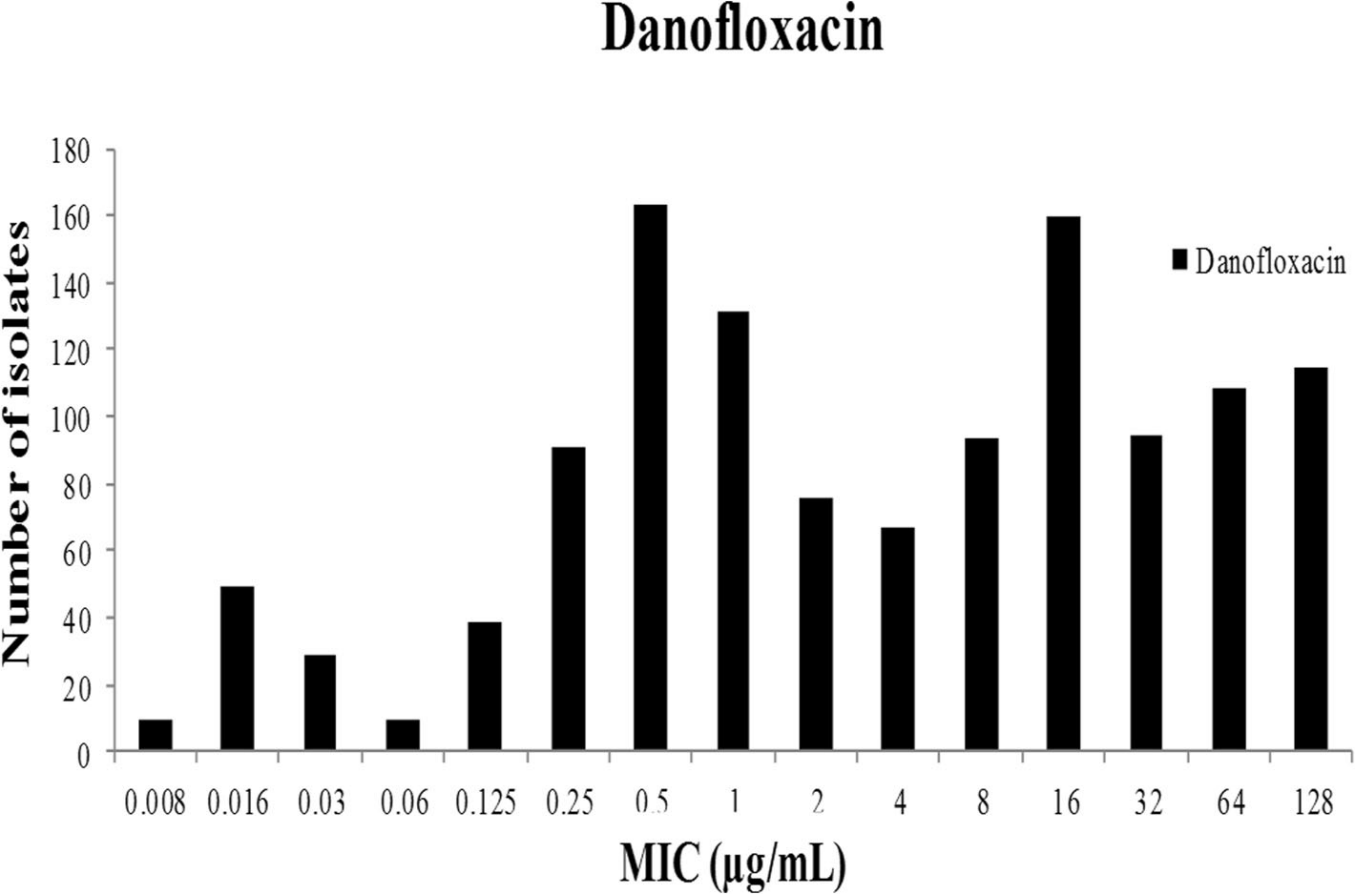
Primary MIC distribution of danofloxacin against 1233 *E.coli* isolates

As shown in the primitive DANO MIC distribution in Fig. 1, MICs for DANO against 1233 *E.coli* isolates (861 isolated, 372 donated) were in the range of 0.008 to 128 μg/mL. The percentages at each MIC (0.008, 0.016, 0.03, 0.06, 0.03, 0.25, 0.5, 1, 2, 4, 8, 16, 32, 64 and 128 μg/mL) were 0.73, 3.97, 2.35, 0.73, 3.16, 7.38, 13.22, 10.62, 6.16, 5.43, 7.54, 12.98, 7.62, 8.76 and 9.33%. The MIC_50_ and MIC_90_ were 4 and 128 μg/mL, respectively.

### Epidemiological cut-off values

**Fig. 2:**
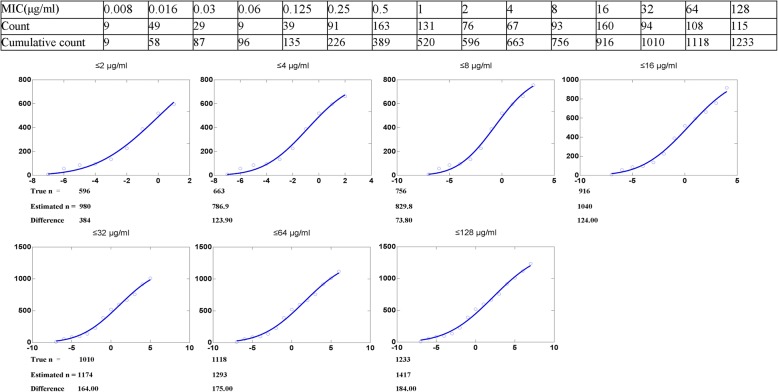
Iterative non-linear regression curve fitting with increasing subsets. X axis = Log_2_ MIC, Y axis = numbers of isolates. Numbers below each graph are the values for the true number of isolates included in the dataset (True n), the non-linear regression estimate (Estimated n) and the difference between these two values of n (Difference). O = observed numbers; solid line = fitted curve

The MIC distribution (0.008-128 μg/mL) for DANO was statistically consistent with a normal distribution because the skewness (− 0.321) and kurtosis (− 0.731) were negative. As the normal (Gaussian) distribution is widely accepted, non-linear regression curve fitting of cumulative log_2_ MIC data was selected as the preferred method for determining the means and standard deviations of MIC distributions. The procedure involved fitting the initial subset and generating estimates for the number of strains in the subset, the mean and the standard deviation (in log). This procedure was repeated by adding to the previous subset each successive column to create the next subset, and repeating the curve-fitting until it was clear that there was a subset where the absolute difference between the true and estimated number of isolates was a minimum. As shown in Table 1 and Fig. 2, the seven subsets examined showed that the subset ‘MIC = 8 μg/ mL’ gave the minimum difference. As a result, the ECV was defined as 8 μg/mL.

**Table 1:** Optimum non-linear least squares regression fitting of pooled MICs (mg/mL) for danafloxacin and *E.coli*

Subset	Number of isolates						Mean MIC (log_2_)				Standard deviation (log_2_)			
fitted	TRUE	Est.	Diff.	ASE	Est./ASE	95%CIb	Est.	ASE	Est./ASE	95%CI^a^	Est.	ASE	Est./ASE	95%CI^b^
≤128	1233.00	1417.00	184.00	106.60	13.29	1185 to 1650	2.22	0.54	4.12	1.046 to 3.394	4.63	0.42	11.01	3.711 to 5.544
≤64	1118.00	1293.00	175.00	108.50	11.92	1054 to 1532	1.64	0.55	2.95	0.4138 to 2.856	4.27	0.43	9.90	3.324 to 5.224
≤32	1010.00	1174.00	164.00	114.30	10.27	919.5 to 1429	1.07	0.59	1.81	−0.2443 to 2.385	3.93	0.46	8.64	2.918 to 4.947
≤16	916.00	1040.00	124.00	113.90	9.13	782.2 to 1298	0.42	0.60	0.70	−0.9379 to 1.780	3.53	0.47	7.53	2.471 to 4.594
≤8b	756.00	829.80	73.80	63.56	13.06	683.2 to 976.3	−0.63	0.36	−1.75	−1.469 to 0.2008	2.81	0.33	8.54	2.051 to 3.569
≤4	663.00	786.90	123.90	93.92	8.38	564.8 to 1009	−0.85	0.51	−1.68	−2.041 to 0.3476	2.66	0.41	6.43	1.679 to 3.634
≤2	596.00	980.00	384.00	353.30	2.77	115.5 to 1844	−0.01	1.47	−0.01	−3.611 to 3.590	3.12	0.79	3.95	1.184 to 5.052

Est., non linear regression estimate of value; Diff., estimate of N minus true N; ASE, asymptotic standard error; Est./ASE, estimate divided by asymptotic standard error

^a^95% CI of estimate of value

^b^This subset gave the smallest difference between the estimate and true number of isolates in the subset

### In vitro time-kill studies

**Fig. 3:**
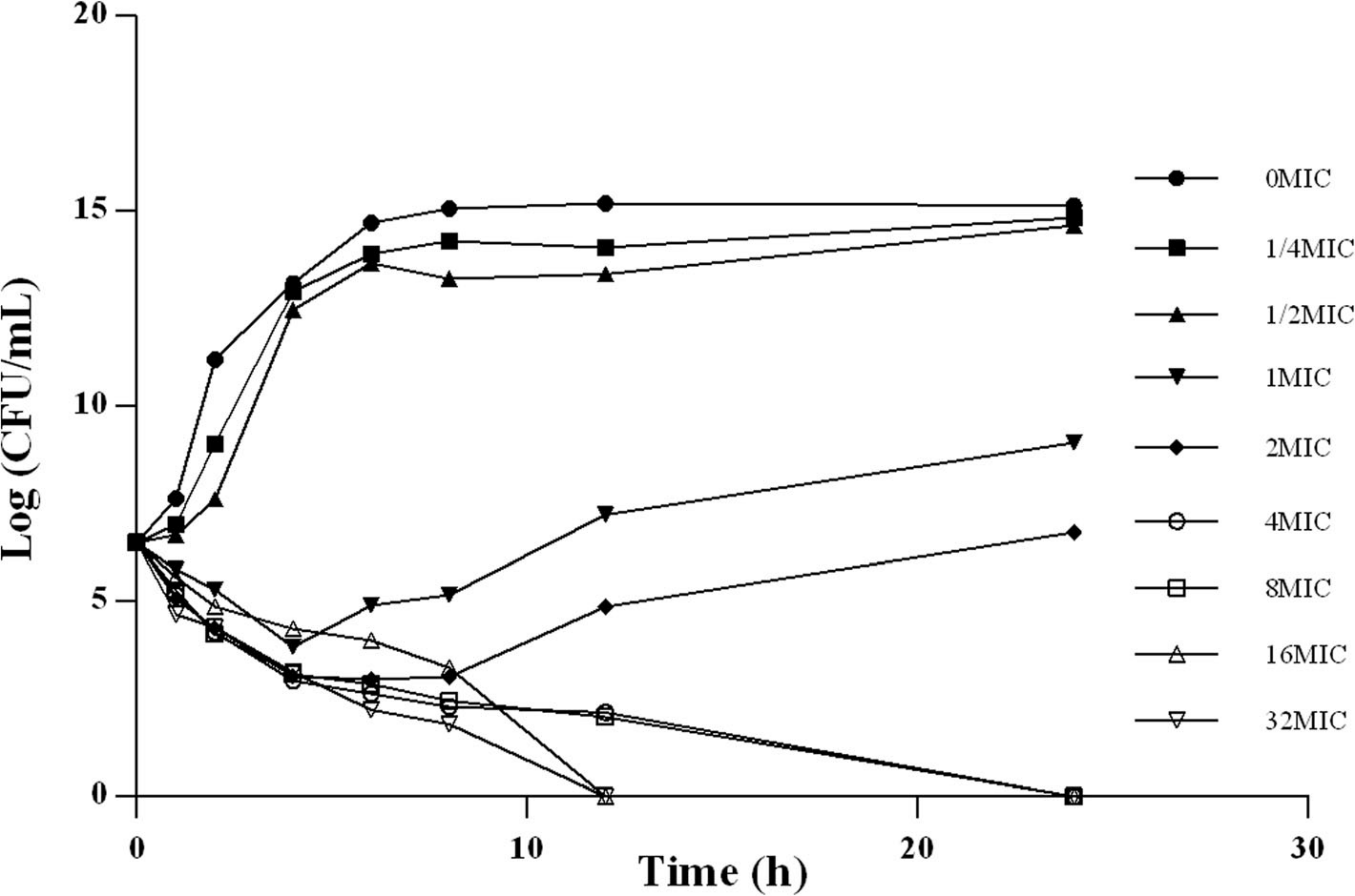
The in vitro time killing curve of danofloxacin against *E.coli*

As presented in Fig. 3, the concentrations (1/4 MIC and 1/2 MIC) below the MIC of DANO can hardly inhibited the growth of *E. coli* JLP95. Similarly, the bacteriostatic effects of 1MIC and 2MIC of DANO are not obvious. However, the antibacterial or bactericidal effects are gradually enhanced when DANO concentrations were at least 4 times higher than MIC. Therefore, the in vitro time-kill test shows that efficacy of DANO against *E.coli* is concentration dependent.

### Pharmacokinetic characteristics of DANO in plasma

**Fig. 4:**
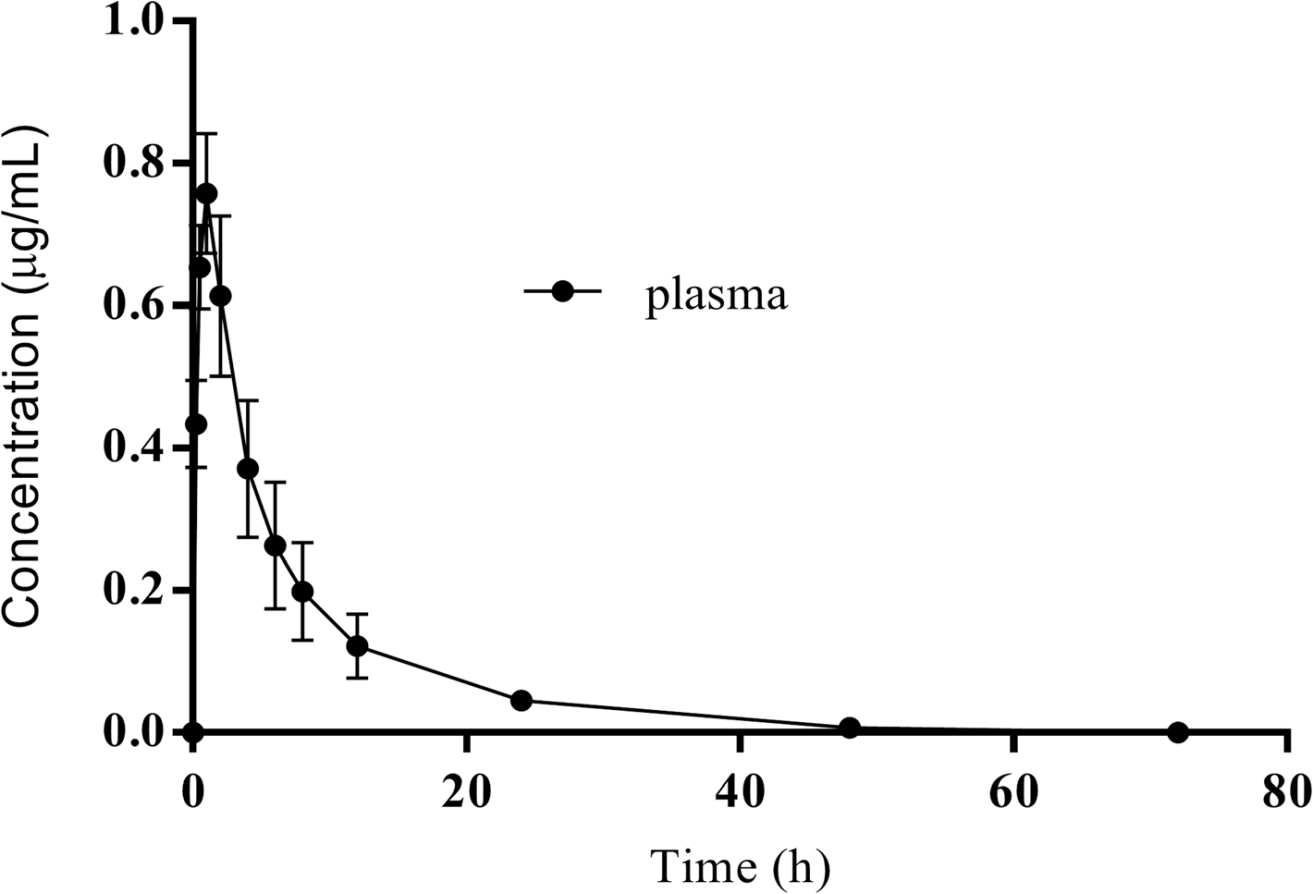
Concentration-time plot of plasma danofloxacin data at 0, 0.25, 0.5, 1, 2, 4, 6, 8, 12, 24, 48, and 72 h after i.m. administration at a dose rate of 2.5 mg/kg in pigs. Values are means±SD (*n* = 6)

There are no adverse reactions after intramuscular injection of DANO. The concentration of plasma DANO was decreased below LOQ after 72 h. The concentration-time curves are presented in Fig. 4. According to MAICE, the plasma data were best fitted to a two-compartmental PK model for all six pigs. Pharmacokinetic parameters are shown in Table 2, the time to reach to maximum drug concentration (T_max_), the peak drug concentration (C_max_), and the area under the curve by 24 h (AUC_0–24_) were 0.97 ± 0.08 h, 0.76 ± 0.08 μg/mL, and 5.25 ± 1.35 h·μg/ml, respectively.

**Table 2:** PK parameters for danofloxacin in pig plasma after i.m. administration at a dose of 2.5 mg/kg (*n* = 6)

PK parameter	Unit	Mean ± SD
A	μg/mL	2.09 ± 1.46
B	μg/mL	0.41 ± 0.17
α	1/h	0.81 ± 0.41
β	1/h	0.10 ± 0.02
K_a_	1/h	2.00 ± 0.51
K_10_	1/h	0.25 ± 0.08
K_12_	1/h	0.32 ± 0.20
K_21_	1/h	0.34 ± 0.23
T_1/2Ka_	h	0.37 ± 0.09
T_1/2α_	h	1.07 ± 0.60
T_1/2β_	h	7.28 ± 1.10
AUC	h*μg/mL	5.25 ± 1.35
T_max_	h	0.97 ± 0.08
C_max_	μg/mL	0.76 ± 0.08
CL	L/h	7.75 ± 1.74
V_c_	liter/kg	2.28 ± 0.39
V_p_	liter/kg	2.16 ± 0.86
V_ss_	liter/kg	4.17 ± 0.76

A and B: Y-axis intercept terms; α: distribution rate constant; β: elimination rate constant; K_a_: absorption rate constant; K_10_: central compartment elimination rate constant; K_12_: rate constant from central to peripheral compartment; K_21_: rate constant from peripheral to central compartment; T_1/2Ka_: absorption half-life of the drug; T_1/2α_: distribution half-life of the drug; T_1/2β_: elimination half-life of the drug; AUC: area under the curve of plasma concentration-time; T_max_: the time point of maximum plasma concentration of the drug; C_max_: the maximum plasma concentration; CL: body clearance; V_c_: volume of distribution in the central compartment; V_p_: volume of distribution; V_ss_: volume of distribution at steady state

### Monte Carlo analysis

**Fig. 5:**
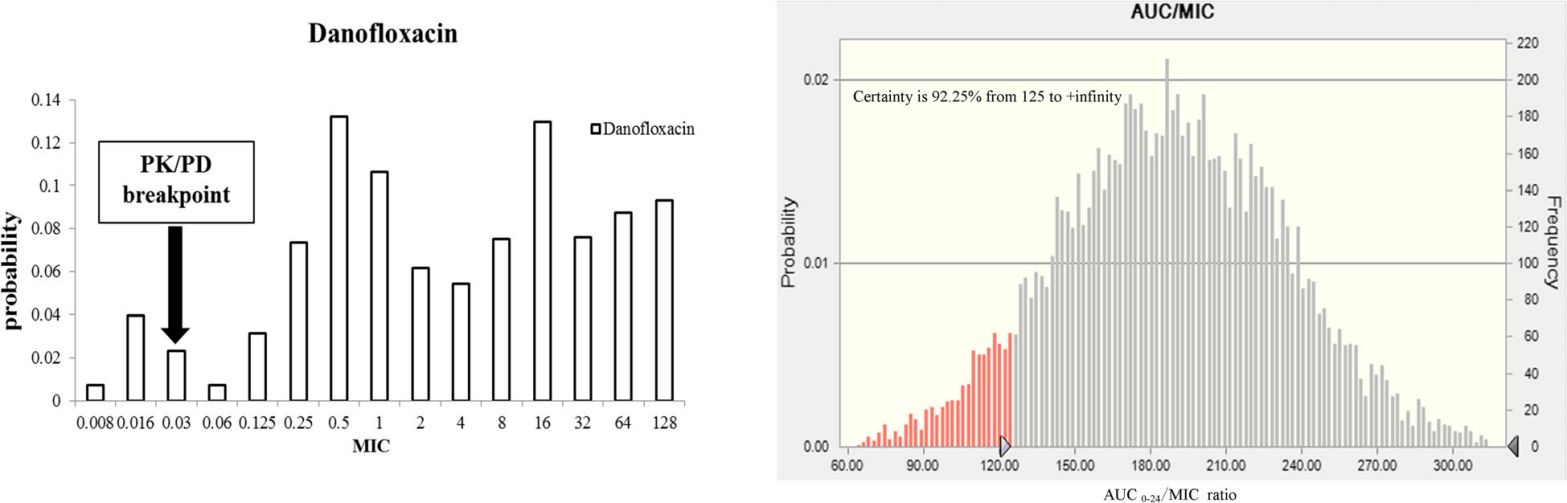
Results of a 10,000-iteration Monte Carlo simulation for danofloxacin based on MIC and AUC_0–24_. The red bars represent the number of simulated with AUC: MIC ratios < 125, whereas the gray bars represent with AUC: MIC ratios of ≥ 125. The probability of danofloxacin attaining an AUC: MIC ratio of at least 125 is 92.25%. Therefore, the CO_PD_ was defined as 0.03 μg/mL

Results of a 10,000- Monte Carlo simulation for DANO based on MIC and AUC_0–24_, the probability of achieving various AUC: MIC ratios at breakpoints of 0.03 μg/mL are presented in Fig. 5. The red bars represent the number of simulated with AUC: MIC ratios < 125, whereas the gray bars represent with AUC: MIC ratios of ≥125. The probability of DANO attaining an AUC: MIC ratio of at least 125 is 92.25%. Therefore, the CO_PD_ was defined at 0.03 μg/mL. The following statistical parameters describe the DANO AUC: MIC probability distribution: mean 188.84, median 188.86, SD 44.91, variance 2017.05, skewness - 0.024, kurtosis 3.03, coeff. of variability 0.24, minimum range 10.42, maximum range 345.06, and mean std. error 0.45.

## Discussion

DANO is a fluoroquinolone antibacterial drug that was developed specifically for veterinary use [23]. However, the resistant *E.coli* isolates are emerging quickly with the use of these drugs [24]. A total of 1737 *E. coli* isolates collected from the United States, Canada, Belgium, France, Germany, Italy, the Netherlands, Spain, the United Kingdom, Japan, and South Africa showed a high degree of susceptibility to DANO, more than 99.3% of the *E. coli* isolates with an MIC of ≤1.0 μg/ml [25]. While, in our study, 50.82% of the *E. coli* isolates (*n* = 1233) with MIC >1.0 μg/ml for DANO, which indicated that temporal and geographic differences could be frequently found on the prevalence of resistance.

Susceptibility breakpoints of quinolones and fluoroquinolones (ciprofloxacin, levofloxacin, cinoxacin, enoxacin, gatifloxacin, gemifloxacin, grepafloxacin, lomefloxacin, nalidixic acid, norfloxacin, ofloxacin, and fleroxacin) for *Enterobacteriaceae* have been established. CLSI [16] recommendations for testing human pathogens against fluoroquinolone, but few guidelines are available for the interpretation of veterinary MIC data. ECV can be used as the most sensitive measurement of the emergence of strains with decreased susceptibility to a given compound when compared with the WT population. These non-WT strains may exhibit one or more acquired resistance mechanisms [26]. Several methods have been reported for determination of ECVs. For instance, Arendrup et al. estimated ECVs as 2-fold dilution steps higher than the MIC_50_ [27], Rodriguez-Tudela et al. estimated them as 2-fold dilutions above the modal MIC [28], and Kronvall and Turnidge et al. calculated ECVs by statistical methods [17, 18]. We used nonlinear least squares regression analysis reported in the CLSI methodology to determine ECVs. Finally, the ECV of *E.coli* for DANO was defined as 8 μg/mL according to the preliminary MIC distribution in our study.

Several studies have established pharmacokinetic data for DANO in a number of farm animal species, including cattle, sheep, goat, chicken and pig [23, 29–32]. The difference in pharmacokinetic parameters between different studies may be due to different breeds or individual differences in pigs. In this study, after i.m. administration of DANO at a dose of 2.5 mg/kg body weight in pigs, the time to reach to maximum concentration (T_max_) was 0.97 ± 0.08 h, similar to 1 h as reported by Mann and Frame, but different from 0.64 h as described by Wang et al.*;* the peak drug concentration (C_max_) was 0.76 ± 0.08 μg/mL, higher than 0.45 ± 0.09 μg/mL, and was half of 1.5 μg/mL reported by Rottboll and Friis which dosing 5.0 mg/kg; the area under the curve by 24 h (AUC_24_) was 5.25 ± 1.35 h•μg/ml, higher than 3.34 ± 0.43 h•μg/ml as reported by Wang et al. [10, 31, 33].

From a pharmacodynamic point of view, fluoroquinolones are considered concentration-dependent rather than time-dependent [34]. The in vitro time kill test in our study demonstrated that DANO is also concentration dependent. Thus, the ratios of peak concentration or AUC with the MIC were reported as important determinants of the antibacterial effect of fluoroquinolones in vitro and the ratio of AUC_24_/MIC or C_max_/MIC were generally used for PK/PD modeling [21, 35].

The parameters C_max_/MIC and AUC_24_/MIC correlate well with therapeutic outcome. A correlation of these composite parameters to the efficacy of an antibacterial revealed that for fluoroquinolones an AUC_0–24_/MIC of ≥125 is predictive of favorable clinical outcome for Gram negative bacteria [21]. The probability of DANO attaining an AUC: MIC ratio of at least 125 is 92.25%. Therefore, the CO_PD_ was defined at 0.03 μg/mL.

To the best of our knowledge, this is the first study to establish the ECV and CO_PD_ of DANO against *E.coli* by statistical method. The CO_PD_ (0.03 μg/mL) was much lower than the ECV (8 μg/mL) established in our study, implying that the lower CO_PD_ in our study may be due to the lower dose of drug administration to pigs, because previous studies concluded that the dose of drug administration may affect the PK-PD breakpoint [36–38]. This suggests that (i) more dosing regimens need to be designed in future investigation; (ii) Further studies are needed to illustrate the relationship between DANO non-WT values proposed in this study and its resistant molecular mechanisms.

## Conclusions

Compared to the CO_PD_ of DANO against *E.coli* (0.03 μg/mL), the ECV for *E.coli* seemed reasonable to be used as the final breakpoint of DANO against *E.coli* in pigs. Therefore, the ECV (MIC≤8 μg/mL) was finally selected as the optimum DANO susceptibility breakpoint for swine *E.coli*, which could provide a criterion for DANO susceptibility testing and improve prudent use of DANO for protecting public health.

## Methods

### Isolates

The rectal/cloacal swabs collected on each farm from the pigs were pooled and tested as one analytical sample. Between July 2014 and March 2017, a total of 864 rectal/cloacal swabs of pig were collected in Heilongjiang (*n* = 297), Jilin (*n* = 151), Liaoning (*n* = 240), Henan (*n* = 97), Shandong (*n* = 30), Hubei (*n* = 20), and Yunnan (*n* = 29) provinces of China. In order to isolate *E. coli*, swabs were streaked out on MacConkey agar plates (Qingdao Hope Bio-Technology Co., Ltd., Qingdao, China) and incubated at 37 °C for 18 to 24 h. The putative *E. coli* isolates on MacConkey agar (bright pink with a dimple) per sample were transferred to eosin methylene blue agar (Qingdao Hope Bio-Technology Co., Ltd., Qingdao, China) for further purification and were incubated at 37 °C for 18 to 24 h. One colony with typical *E. coli* morphology was selected from each sample and identified by conventional biochemical methods according to ‘Bergey’s Manual of Determinative Bacteriology’. In addition, a total of 372 *E.coli* strains were respectively donated by National Key Laboratory of Veterinary Biotechnology, Harbin Veterinary Research Institute, Chinese Academy of Agricultural Sciences (*n* = 108), Husbandry and Veterinary College, Jilin University (*n* = 112), and College of Animal Husbandry and Veterinary Science, Henan Agricultural University (*n* = 152). All of the bacterial isolates were confirmed by polymerase chain reaction (PCR) [39].

### Antibacterial susceptibility testing

Broth microdilution testing was performed in accordance with the guidelines in CLSI document M07-A9 [40] at the following laboratories: Department of Microbiology, Department of Pharmacology and Toxicology, and Pharmacy Department in Northeast Agricultural University, Harbin, China. Pure powder of DANO (Qingdao Hope Bio-Technology Co., Ltd., Qingdao, China) was dissolved in ultrapure water to prepare stock solutions of 5120 μg/mL. Two-fold serial drug dilutions were prepared in broth (Qingdao Hope Bio-Technology Co., Ltd., Qingdao, China) to achieve the final concentration ranged from 0.008 to 128 μg/mL. Each well contains approximately 5 × 10^5^ CFU/mL *E.coli* and cultured in 96 well plates. Plates were incubated at 37 °C for 20 h. Quality control (QC) isolate *E.coli* ATCC 25922 was used on each day of testing by the participating laboratories, as recommended by CLSI [40]. Only those results, for which the QC MICs were within the established reference range, were used in the study. The MIC is the lowest concentration of antimicrobial agent that completely inhibits growth of the organism in the microdilution wells as detected by the unaided eye. All MIC determinations were performed in triplicate.

### Definitions

The ECV (also known as the wild-type cutoff, or CO_WT_), defined as the highest susceptibility endpoint of the wild-type (WT) population MIC, has been shown to detect the emergence of in vitro resistance or to separate WT isolates (without known mechanisms of resistance) from non-WT isolates (with mechanisms of resistance and reduced susceptibilities to the antibacterial agent being evaluated) [26, 41]. ECVs are calculated by taking into account the MIC distribution, the modal MIC of each distribution, and the inherent variability of the test (usually within one doubling dilution) and should encompass ≥95% of isolates [17].

### Analysis

To analyze the MIC distributions, MICs were transformed into log_2_ values. The skewness and kurtosis of each MIC distribution were determined. Skewness quantifies the degree of symmetry of the distribution, whereas kurtosis quantifies the extent to which the shape of the data distribution matches the normal distribution. To confirm the presence of more than one MIC distribution, frequency distributions of MIC data were analyzed by nonlinear least squares regression analysis based on the following Cumulative Gaussian Counts equation:Z = (*X* − *Mean*)/*SD*, Y = N ∗ zdist(*z*), in which the Mean is the average of the original distribution, from which the frequency distribution was created; SD is the standard deviation of the original distribution (calculations were performed using Prism 6.0 software, San Diego, CA). Three parameters were estimated, the mean and SD (both log_2_), and the total number (N) in the presumed unimodal distribution. N was estimated rather than taken as a constant in the regression, because of the desire to fit the data to the distribution without assuming that N truly contained only wild-type isolates [17, 42].

### In vitro time-kill studies

In vitro time-kill studies were conducted in Mueller-Hinton broth with concentrations of DANO ranging from 1/4 to 32 times of the MICs for *E.coli* JLP95 (O_8_), which were tested separately. The initial inoculum sizes of the bacteria used to generate the time-kill profiles were approximately 10^6^ CFU/mL. 1.2 mL of co-culture was removed from each tube and then 200 μL of co-culture was continuously diluted with 1.8 mL Mueller-Hinton broth to measure the CFU at 0, 1, 2, 4, 6, 8, 12 and 24 h following inoculation.

### Animals

Six 5-months-old healthy fragrance pigs weighing 14–16 kg were donated from Clinical Surgery Department’s pig breeding farm of Northeast Agricultural University for free use. All animals were provided with a drug-free commercial diet to acclimatize for 1 week prior to the study. After the trial, all animals were returned to them for further feeding and used for subsequent laparoscopic trials. All experimental work was performed in accordance with the animal ethics guidelines approved by the animal care and ethics committee of Northeast Agricultural University (Heilongjiang Province PR China).

### Pharmacokinetic study

DANO (purity > 99%) was obtained from Zhejiang Guobang Pharmaceutical Company Limited, China. The pure reference standard of DANO was obtained from the Sigma-Aldrich (China).

DANO was intramuscularly injected at 2.5 mg DANO/kg body weight in each pig. Blood samples (5 mL) from the brachiocephalic vein were collected into EDTA dipotassium salt tubes at 0.15, 0.5, 1, 2, 4, 6, 8, 12, 24, 48 and 72 h after injection. Plasma was separated by centrifugation at 3000 g for 10 min and stored at − 20 °C until analysis.

The method for the analysis of DANO concentration in plasma was modified from that described by [43, 44]. The HPLC system Waters 2695 was connected to a Waters 2475 fluorescence detector (λex =280 nm and λem = 450 nm) with a mixture of acetonitrile and aqueous solution (15:85, *v*/v) as the mobile phase. The aqueous solutions were prepared by dissolving potassium dihydrogenophosphate (0.020 M), phosphoric acid (0.006 M), and tetraethylammonium bromide (0.012 M) in water. The pH of the mobile phase was adjusted to 3.0 by addition of 2 N NaOH. The flow rate was set at 1.0 mL/min; A Waters C_18_ reverse phase column C_18_ (250 mm × 4.6 mm I.D.; particle size, 5 μm) was used to perform HPLC at 30 °C; and the injection volume was 10 μL.

Samples were thawed at room temperature, and 10 μL of 50 μg/mL ciprofloxacin (Sigma–Aldrich) was added to plasma (0.5 mL) as the internal standard. After adding 3 mL of acetonitrile, the mixed samples were shaken at 220 oscillations/min for 15 min and then centrifuged at 12000 g for 10 min. The organic layer was transferred into a sterilized tube and dried at 40 °C under nitrogen stream. The residue was dissolved in the mobile phase (0.5 mL), and 10 μL injected for HPLC analysis.

The limit of detection (LOD) was 0.005 μg/mL and the limit of quantification (LOQ) was 0.01 μg/mL in plasma, respectively. Standard curves were linear from 0.01 to 1.5 μg/mL in plasma (*R*^2^ = 0.9999). The inter-day variation for determination in plasma ranged from 0.18 to 1.50%. The recovery of DANO in plasma ranged from 85.80 ± 0.16% to 103.40 ± 4.89%.

### Pharmacokinetic analysis

PK analysis was conducted by using WinNonlin v.5.2.1 (Pharsight Corporation, Mountain View, CA, USA). Mininmum Akaike Information Criteria Estimates (MAICE) was applied to determine the best fit of model for each pig [45].

### Monte Carlo analysis

A 10,000-subject Monte Carlo simulation was conducted for each drug at each of the following MIC: 0.008, 0.016, 0.03, 0.06, 0.03, 0.5, 1, 2, 4, 8, 16, 32, 64 and 128 μg/mL using ORACLE CRYSTAL BALL software (version 11.1; Oracle USA, Denver, CO, USA). Based on pharmacokinetic results of DANO in pigs in this study, a conservative PK/PD value (AUC_0–24_/MIC = 125) was selected to calculate the PTA [21]. AUC_0–24_ was not measured for the fluoroquinolones, it was calculated as follows: AUC_0–24_ = Dose/V_ss_ × K_d_, where V_ss_ was the volume of distribution at steady state (L/kg) and K_d_ the elimination rate constant (h^− 1^) [20]. AUC_0–24_ was assumed to be log-normally distributed, and the PK/PD indices were calculated for each simulated subject. The PTA was estimated at each MIC as the probability that at least the target level of the PK/PD index is achieved. The CO_PD_ was defined as the highest MIC at which the PTA was ≥90% [46, 47].

## Abbreviations

CLSI: Clinical and Laboratory Standards Institute
CO_PD_: Pharmacodynamic cutoff
DANO: Danofloxacin
*E.coli*: *Escherichia coli*
ECV: Epidemiologic cutoff value
ETEC: Enterotoxigenic *E. coli*
MICs: Minimum inhibitory concentrations
PK: Pharmacokinetic
PTA: Probability of target attainment
PWD: Postweaning diarrhea
WT: Wild type
